# BAT3 Guides Misfolded Glycoproteins Out of the Endoplasmic Reticulum

**DOI:** 10.1371/journal.pone.0028542

**Published:** 2011-12-08

**Authors:** Jasper H. L. Claessen, Hidde L. Ploegh

**Affiliations:** Whitehead Institute for Biomedical Research, Department of Biology, Massachusetts Institute of Technology, Cambridge, Massachusetts; International Centre for Genetic Engineering and Biotechnology, Italy

## Abstract

Secretory and membrane proteins that fail to acquire their native conformation within the lumen of the Endoplasmic Reticulum (ER) are usually targeted for ubiquitin-dependent degradation by the proteasome. How partially folded polypeptides are kept from aggregation once ejected from the ER into the cytosol is not known. We show that BAT3, a cytosolic chaperone, is recruited to the site of dislocation through its interaction with Derlin2. Furthermore, we observe cytoplasmic BAT3 in a complex with a polypeptide that originates in the ER as a glycoprotein, an interaction that depends on the cytosolic disposition of both, visualized even in the absence of proteasomal inhibition. Cells depleted of BAT3 fail to degrade an established dislocation substrate. We thus implicate a cytosolic chaperone as an active participant in the dislocation of ER glycoproteins.

## Introduction

Protein folding in the endoplasmic reticulum (ER) is an inherently fallible process. Terminally misfolded ER glycoproteins leave the folding cycle and are often targeted for dislocation to the cytosol, followed by ubiquitin-dependent degradation by the proteasome (reviewed in [1]). Many ER luminal proteins have been identified that are involved in shuttling misfolded polypeptides to the hypothesized dislocon, the identity and composition of which remain to be defined more fully in molecular terms. BiP, OS-9, XTB3-B, PDI, and members of the EDEM family are thought to target the polypeptide to the dislocon [1]. At the same time they help maintain solubility to prevent detrimental build-up of aggregated, misfolded translation products inside the ER lumen [2].

Unfolded polypeptides interact with chaperones to prevent exposure of hydrophobic amino acids or putative transmembrane domains prior to the completion of protein folding or membrane insertion, cotranslational protein translocation into the ER being a prime example [3]. Misfolded ER glycoproteins exit from the ER in a process called dislocation (or retrotranslocation) [4], [5]. Although different modes of escape have been proposed, a conserved dislocation reaction that involves poly-ubiquitylation, followed by extraction by the dedicated AAA ATPase p97, operates in both yeast and mammals [6], [7], [8]. Misfolded proteins that undergo dislocation almost certainly display features of a partly unfolded polypeptide. The definition of dimensional restrictions imposed by the putative dislocon must await its more complete molecular characterization. In any case, polypeptides that are fed into p97 AAA ATPase, as well as those entering the proteolytic core of the proteasome require complete unfolding [9], [10], [11].

How the cell avoids aggregation of ER glycoproteins discharged into the cytosol is poorly understood. When proteasomal proteolysis is blocked, a soluble version of Class I MHC products -bona-fide type I membrane proteins- occurs in the cytoplasm of cells that express viral immunoevasins, with the transmembrane segment, normally inserted into a lipid bilayer, fully intact [4], [5]. Tight coupling between proteasomal proteolysis and dislocation may prevent exposure of transmembrane segments to an aqueous environment, or perhaps chaperones temporarily shield such segments until the proteasome (or an other protease) can be engaged.

We previously showed that expression of a highly active, Epstein-Barr virus-derived deubiquitylating enzyme domain (EBV-DUB) blocks proteasomal degradation of cytosolic and ER-derived proteins by preemptive removal of ubiquitin from proteasome substrates [12]. Upon EBV-DUB expression, a misfolded ER glycoprotein accumulates in association with the cytosolic chaperone BAT3 (BAG6/Scythe) as a deglycosylated cytosolic intermediate [12].

We now place BAT3 in the extraction pathway for ER glycoproteins. We find that it associates with Derlin2 at the ER membrane and engages misfolded ER proteins, whereas depletion of BAT3 hampers their degradation. We thus assign a role for a cytosolic chaperone in the dislocation reaction. While these experiments were in progress, Ye and colleagues [13] published data fully consistent with the earlier proposal that cytosolic chaperones associate with cytoplasmic dislocation products [12] and our observations agree with this report [13].

## Results

### BAT3 associates with Derlin2

Dislocation and degradation of ER glycoproteins can be uncoupled by expression of a highly active DUB. The misfolded ER luminal glycoprotein truncated Ribophorin I (Ri332) accumulates in the cytosol as a deglycosylated dislocation intermediate when expressed together with the EBV-DUB [12]. Because this deglycosylated species accumulated in association with BAT3, we investigated whether BAT3 merely serves as a buffer for dislocation substrates stalled in the cytoplasm to maintain them in disaggregated form, or whether BAT3 plays an active role in the dislocation reaction itself.

**Figure 1:**
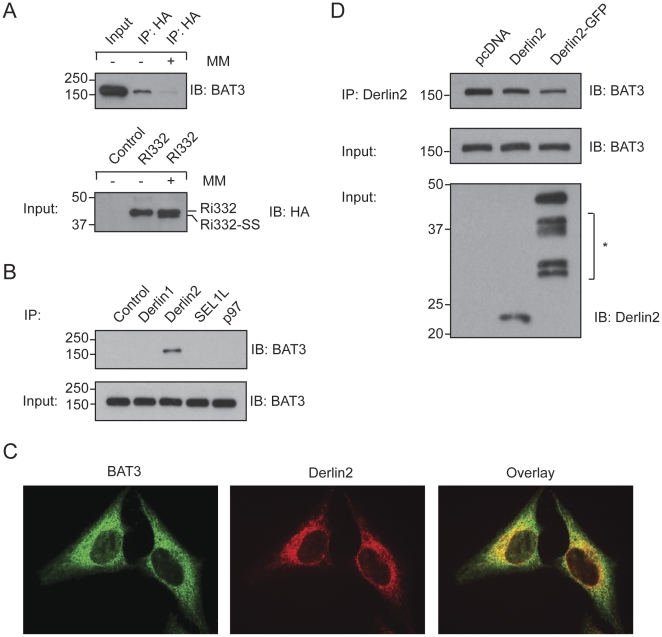
BAT3 associates with Derlin2. A. HA-Ri332 was synthesized in a rabbit reticulocyte lysate in the presence or absence of canine pancreatic microsomal membranes (MM). Following NP40-mediated lysis, Ri332 was retrieved by immunoprecipitation. The immunoprecipitate and input samples were blotted for either BAT3 or HA as indicated. B. 293T cells were subjected to NP40 lysis, followed by retrieval of the indicated proteins. Pre-immune serum served as a control. The eluates were blotted for BAT3, as were the input control samples. C. Immunofluorescence of Hela cells using antibodies against endogenous BAT3 (green) and Derlin2 (red). Scale bar = 5 µm. D. 293T cells were transiently transfected with the indicated constructs, subjected to NP40 lysis followed by an immunoprecipitation for Derlin2. Immunoprecipitates were blotted for BAT3. Input samples were blotted for BAT3 and Derlin2. The *asterisk* indicates non-specific polypeptides.

To validate the observed interaction, we first reproduced it in an *in vitro* setting. Ri332 was synthesized in an *in vitro* translation system and retrieved by an immunoprecipitation reaction after mild lysis. BAT3 was readily retrieved in complex with Ri332, but this interaction was not observed when translation was carried out in the presence of microsomal membranes (Figure 1A). Under these conditions Ri332 was translocated into the lumen of the microsomes, as indicated by cleavage of its signal sequence. This indicates that the observed interaction between Ri332 and BAT3 depends on the cytosolic disposition of both interacting partners.

BAT3 could still be involved in the dislocation reaction when in close proximity to the ER membrane, because at least some of the dislocation intermediate observed upon expression of the EBV-DUB remains loosely associated with the ER membrane [12]. We therefore examined whether BAT3 engages any of the known dislocation components that localize to the ER membrane. We readily retrieved BAT3 in association with Derlin2, a small membrane protein implicated in ER quality control (Figure 1B) [14], [15].

Although BAT3 is reported to localize to the nucleus [16], we demonstrated additional co-localization with Derlin2 by immunofluorescence microscopy (Figure 1C), in line with assigned roles for BAT3 in the cytosol [13], [17]. In order to characterize this interaction further, we examined the consequences of overexpression of Derlin2. The stoichiometry of multi-protein complexes can be disturbed by overexpression of one of its components. We recovered a reduced amount of BAT3 upon overexpression of Derlin2, and we observed an even more obvious reduction when we overexpressed a Derlin2-GFP fusion protein (Figure 1D). The latter effect we ascribe to the bulk of the appended GFP moiety, which would sterically hinder these interactions.

We conclude that BAT3 can interact with a dislocation substrate and that it is recruited to the site of dislocation through interactions with Derlin2, although our data do not distinguish between direct and indirect interactions. Of note, members of the Derlin family have been speculated to form a channel that facilitates passage of misfolded polypeptides from the lumen of the ER across the ER membrane to the cytoplasm [18], [19].

### Visualizing a dislocation complex

Having shown interactions between BAT3 and Ri332, and between BAT3 and Derlin2, we set out to visualize these complexes by microscopy. As Ri332 is rapidly destroyed, we reasoned that we could best visualize any such complex by inhibiting degradation. To this end, we interfered with degradation by expression of a dominant negative version of YOD1 (YOD1 C160S) [20] or the EBV protease domain targeted to p97 (UBX-EBV) [12].

Hela cells were transiently transfected with Ri332, upon which the various types of blockade were imposed. After fixation, cells were permeabilized and triple-labeled for Ri332 (HA), Derlin2 and BAT3. We directed our attention to cells with a strong signal for the dislocation substrate, as an indication of successful inhibition of degradation.

**Figure 2:**
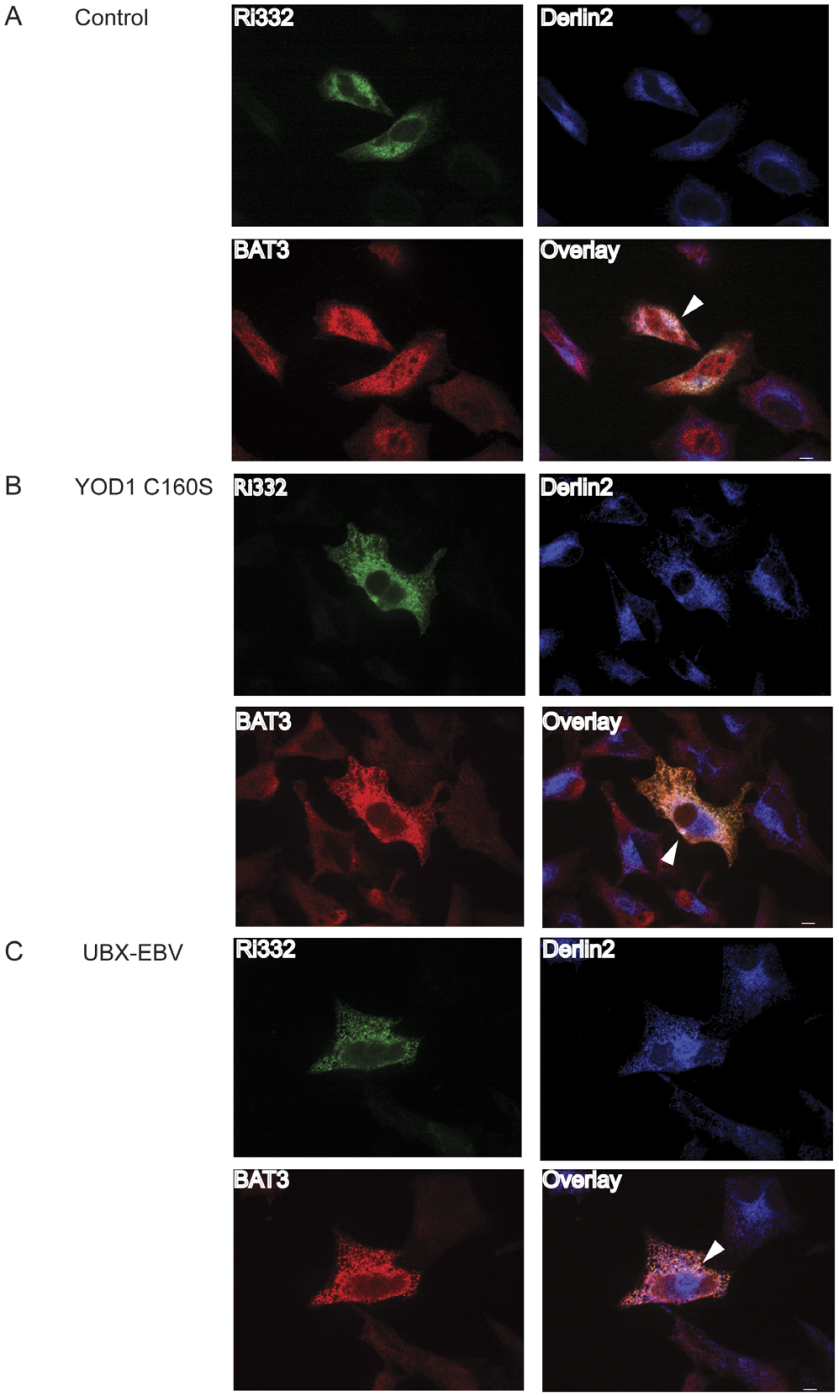
BAT3 localizes to a complex with Derlin2 and Ri332. Hela cells were plated on coverslips and transiently transfected with HA-Ri332 and empty vector (A), YOD1 C160S (B) or UBX-EBV (C). After paraformaldehyde fixation, cells were labeled for HA (green), BAT3 (red), and Derlin2 (blue). Scale bar = 5 µm.

In control cells, we observed clear co-localization of Ri332, BAT3 and Derlin2 (white color) consistent with the notion that BAT3 can engage a misfolded ER luminal glycoprotein at the site of dislocation (Figure 2A).

Irrespective of the type of blockade imposed, we see near complete co-localization between Ri332 and BAT3 (Figure 2B, 2C). Ri332 is thought to accumulate partly if not mostly inside the ER lumen when degradation is blocked. However, if stalled dislocation substrates were to accumulate at the luminal site of dislocation, such localization would result in the observed staining pattern. Under conditions of ongoing proteasomal proteolysis, Ri332 does not accumulate in such complexes, because its levels rapidly drop due to proteasomal activity. We conclude that BAT3 localizes to a dislocation substrate at the site of a dedicated dislocation component.

### BAT3 is required for dislocation of TCRα

**Figure 3:**
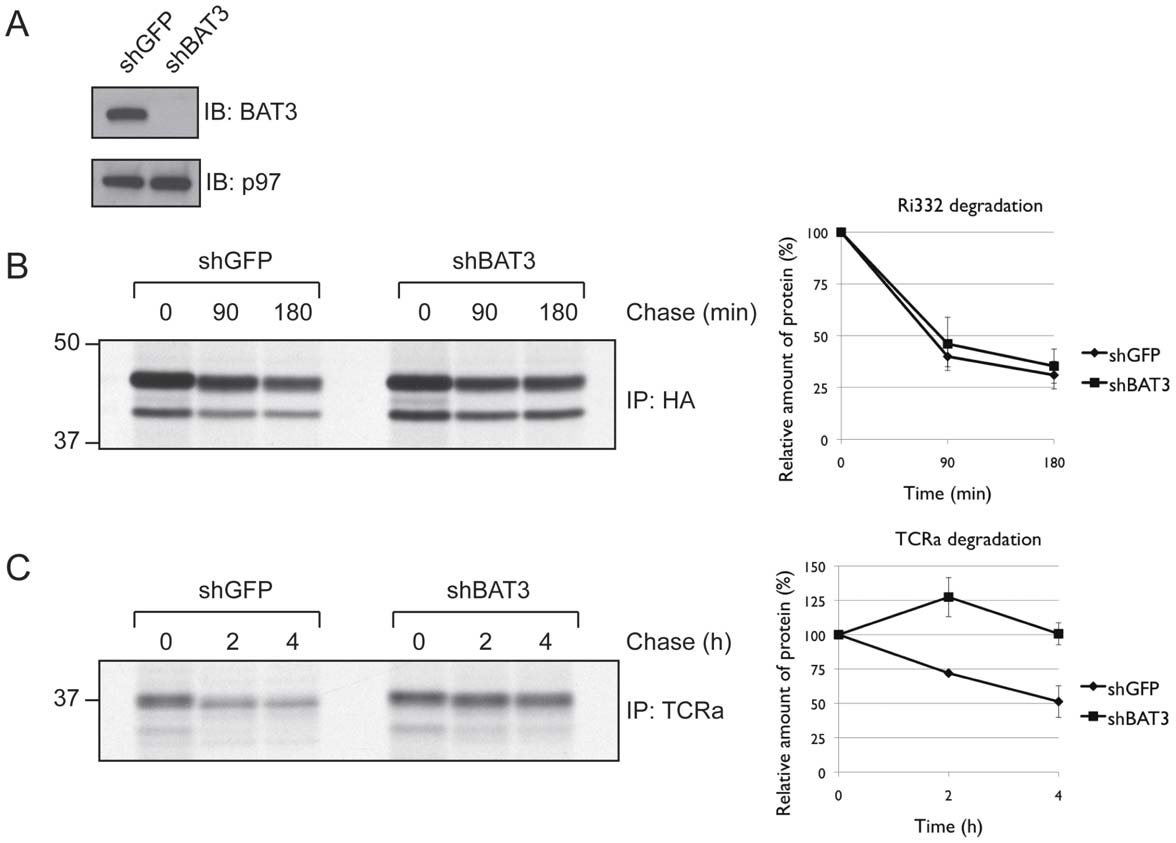
BAT3 is required for dislocation of TCRα. A. 293T were stably transduced with short RNA hairpins against either GFP or BAT3. SDS lysates were immunoblotted for either BAT3 or p97. B. BAT3 knock-down cells (a) were transiently transfected with HA-Ri332 and subjected to pulse-chase analysis. Densitometric quantitation of the relative amount of protein is shown (n = 9). C. As in (b), except that cells were transfected with TCRα (n = 3).

Having shown that BAT3 can associate with a misfolded, dislocated ER glycoprotein in vitro and that BAT3 can occur in a complex with both the degradation substrate and Derlin2 when proteasomal degradation is inhibited, we examined whether there was a direct requirement for BAT3 in the dislocation reaction itself. We performed a BAT3 knock-down in 293T cells through stable expression of a short RNA hairpin against BAT3 (Figure 3A).

Despite the strong knock-down seen for BAT3, we observed at best a modest effect on dislocation of Ri332, as assessed by pulse-chase analysis (Figure 3B). This by no means excludes a role for BAT3 in dislocation of Ri332, as redundancy through the existence of multiple chaperone complexes is not unlikely. The observed effect on Ri332 stands in sharp contrast to the effect observed when we tested the role of BAT3 in dislocation of the alpha chain of the T-cell receptor (TCRα), which undergoes rapid degradation when expressed in the absence of its usual receptor partner subunits. Knock-down of BAT3 resulted in near complete stabilization of TCRα in a pulse-chase experiment (Figure 3C).

Of interest, TCRα carries four N-linked glycans, which are rapidly removed by cytoplasmic peptide N-glycanase when it is successfully discharged in the cytosol [21]. As TCRα accumulates predominantly as the fully glycosylated version, the absence of BAT3 hampers the active removal of TCRα from the ER membrane.

### Disrupted BAT3 binding by Derlin2 slows TCRα degradation

**Figure 4:**
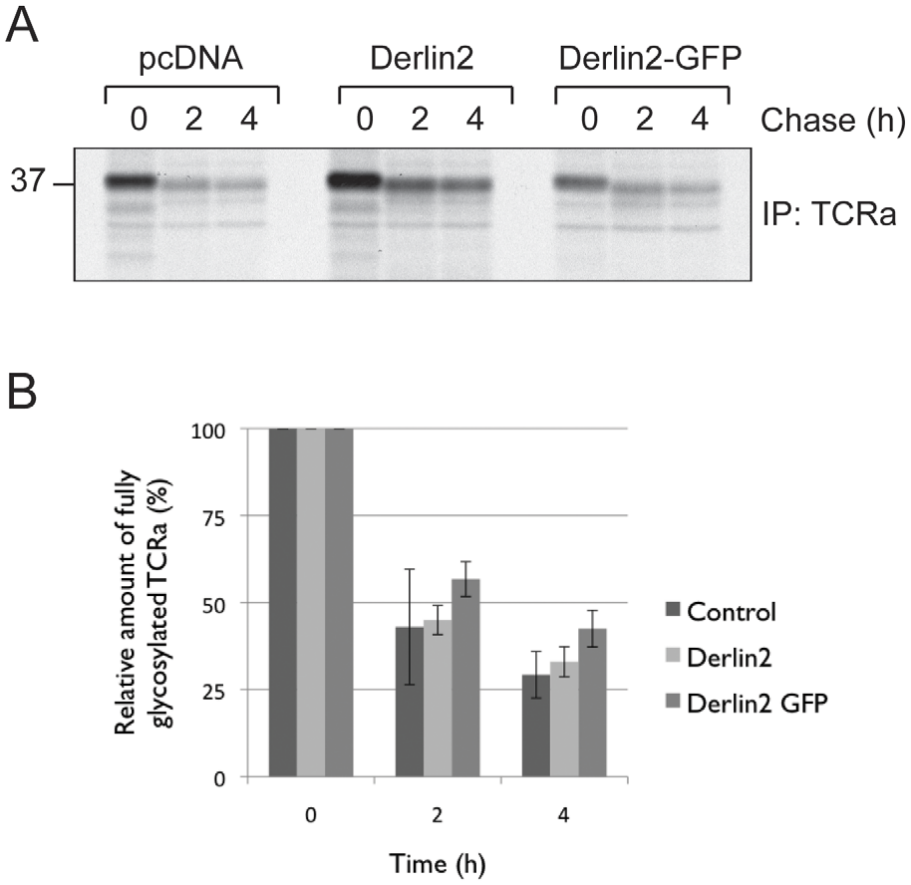
Impaired BAT3 recruitment to Derlin2 slows dislocation. A. 293T cells were transiently transfected with TCRα and either pcDNA, Derlin2, or Derlin2-GFP (see figure 1D). TCR degradation was assessed by pulse-chase analysis. B. Densitometric quantitation of the relative amount of protein is shown (n = 4).

Having established a role for BAT3 in the degradation of TCRα, we wondered whether this could be correlated with its recruitment by Derlin2. As overexpression of Derlin2, and more so Derlin2-GFP, frustrated its interaction with BAT3, (Figure 1D) we tested whether this had any consequences for TCRα degradation. TCRα degradation is reduced in cells that overexpress Derlin2-GFP (Figure 4). The stabilization we observed is modest but in agreement with the continued, but reduced, binding of BAT3 to Derlin2-GFP we discussed above. Members of the Derlin family can form homo- and hetero-dimers, which could overcome, at least in part, the steric hindrance imposed by the bulky GFP molecule used as fusion partner [14].

### TCRα is engaged by BAT3

Can we capture directly the interaction between BAT3 and TCRα? When degradation is allowed to proceed unperturbed, dislocation is tightly coupled to degradation of the substrate, making this a rather transient interaction. We transiently transfected 293T cells with TCRα and labeled them to steady state with [^35^S] cysteine/methionine (overnight labeling). The lysate was precleared with pre-immune serum, followed by immunoprecipitation for TCRα to retrieve any interacting proteins. The immunoprecipitate was then dissolved in 1% SDS at 37 degrees Celsius and subjected to a second round of immunoprecipitation to interrogate the complex for the presence of the indicated proteins.

**Figure 5:**
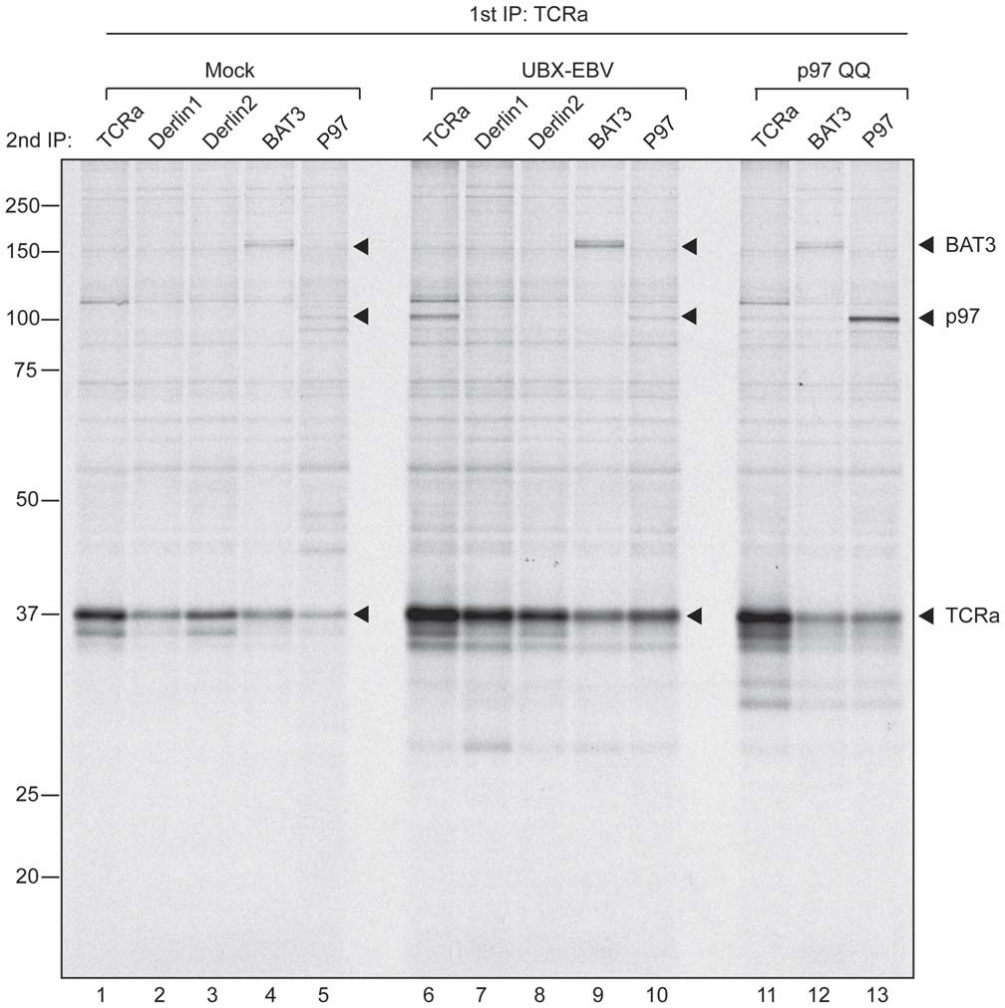
TCRα is engaged by BAT3. 293T cells were transiently transfected with TCRα and either empty vector, UBX-EBV, or p97 QQ, and labeled overnight with [^35^S] methionine/cysteine to achieve steady state labeling. Cells were harvested and subjected to NP40 lysis. The lysates were precleared using pre-immune serum and immobilized protein A. Lysates were adjusted for total levels of incorporated isotope and subjected to immunoprecipitation for TCRα. The captured protein was eluted in 1% SDS at 37°C followed by a second immunoprecipitation for the indicated proteins.

We could retrieve endogenous BAT3 from the TCRα immunoprecipitate, thus visualizing this transient interaction (Figure 5, lane 4). The visualized TCRα (lane 1) does not represent the population that can interact with BAT3, as the indicated polypeptide for TCRα represents fully glycosylated and thus ER-localized TCRα. It is likely that the dislocated population of TCRα escapes detection due to low signal levels or as a consequence of the sequential immunoprecipitation. In addition to BAT3, we also retrieved p97 in complex with TCRα (lane 5). This hexameric AAA ATPase is thought to provide the mechanical force that extracts proteins from the ER and it has been implicated in the dislocation of TCRα, thus validating the retrieval of BAT3 [11].

As a control, we performed the same experiment in cells that were co-transfected with TCRα and either UBX-EBV or p97 QQ. We showed previously that co-expression of UBX-EBV stabilizes the degradation of TCRα and leads to the accumulation of a deglycosylated dislocation intermediate [12]. In line with this observation, we retrieve more TCRα overall (lane 6 versus 1) and more BAT3 specifically (lane 9 versus 4). The p97 QQ mutations render inactive the ATPase activity of the protein, but still allow it to engage a substrate [22]. Accordingly, when we co-expressed p97 QQ we retrieved more TCRα overall (lane 11 versus 1) and more p97 specifically (lane 13 versus 5). Co-expression of UBX-EBV as well as p97 QQ results in higher levels of TCRα, as either membrane extraction, or delivery to the proteasome is blocked. However, only when TCRα is co-expressed with UBX-EBV do we retrieve more endogenous BAT3, ruling out a post-lysis interaction.

We note that two BAT3 species of distinct molecular weight are recovered in association with TCRα. Several isoforms of the protein have been annotated, and we retrieve at least two of them in association with a dislocation substrate.

## Discussion

Proteins expelled from the ER by dislocation are likely to be at least partially unfolded, although an assessment of their true conformational state remains an obvious challenge. Unless tight coupling exists between dislocation and degradation, these (partly) unfolded polypeptides are exposed to an aqueous environment and are prone to aggregation. The latter applies *a fortiori* when considering membrane proteins such as Class I MHC products, one of the few examples for which there is evidence of a soluble cytoplasmic intermediate with its TM segment present and intact [4]. We show here that the cytoplasmic chaperone BAT3 is recruited to Derlin2 at the ER membrane, can engage ER glycoproteins that are subject to dislocation, and that BAT3 is required for dislocation of TCRα.

A disruption of proteasomal degradation not only halts breakdown of ER-derived proteins, but often leads to their accumulation within the ER lumen. This is complemented by the requirement for poly-ubiquitylation to complete the dislocation reaction, giving rise to the idea that dislocation of a substrate is mostly tightly coupled to its degradation. Examples exist where such coupling is undone, for instance the Human Cytomegalovirus proteins US11 and US2, both of which funnel Class I MHC heavy chains into a dislocation pathway, and result in accumulation of the heavy chain in the cytoplasm when proteasomal proteolysis is blocked. We showed that in the presence of the EBV-DUB, ER-derived glycoproteins accumulate in the cytoplasm but are not degraded [12]. Even though these ER proteins accumulate in the cytoplasm, it is unclear how they are maintained in a soluble state once there.

The cytoplasm contains folding machinery that prevents protein aggregation. HSC70 and HSP90, and their heat-shock induced counterparts, are examples of chaperones that engage (partly) unfolded polypeptides in the cytoplasm. Even though these chaperones have been linked to ER protein maturation and dislocation (reviewed in [23]), particularly for the example of HSC70 and its involvement in dislocation of the misfolded cystic fibrosis transmembrane conductance regulator ΔF508 [24], it remains to be established whether they play a direct role in dislocation and bridge the gap between the chaperone-rich ER lumen and the proteasome.

BAT3 has a role in the degradation of defective polypeptides synthesized at the ribosome, and may shuttle such substrates to the proteasome [25], [26]. We now show that BAT3 engages defective ER proteins discharged from the ER, and that BAT3 and its interactors accumulate as a complex when proteasomeal targeting is blocked by expression of the EBV-DUB.

Derlin2 is a small ER membrane protein. Members of the Derlin family have all been implicated in dislocation and may form part of a putative channel (dislocon) that facilitates the passage of misfolded ER proteins to the cytoplasm [14], [15], [18], [19]. We now find that BAT3 interacts with Derlin2 and associates with at least a select set of misfolded proteins. BAT3 is at the nucleus of a protein complex that shields the hydrophobic transmembrane domain of tail-anchored (TA) proteins after ribosomal synthesis and prior to engagement by a dedicated ER-targeting chaperone [17], [27]. BAT3 could perform a similar task for unfolded ER proteins at a dislocation channel, especially when such substrates contain a hydrophobic transmembrane stretch. Similarly, BAT3 was found to sequester mis-targeted proteins from the translocon [26].

Upon depletion of BAT3, the dislocation substrate TCRα accumulates primarily inside the ER lumen (Figure 3C). As BAT3 can function as a co-chaperone recruiter, the interactions we observe most likely occur in the context of a multi-protein complex. In fact, the BAT3-nucleated complex responsible for capturing TA proteins fresh off the ribosome has recently been implicated in protein dislocation [13].

Even though Derlin2 occurs in a complex with both Derlin1 and SEL1L [14], we retrieve BAT3 exclusively with Derlin2 in our immunoprecipitation experiments (Figure 1B). The choice of detergent may contribute to this selectivity: BAT3 may well associate with other components of the dislocation machinery, or the complex may simply not be sufficiently stable to allow retrieval of all of its constituents.

There are two moments during the dislocation process at which a chaperone like BAT3 could be of importance. The first moment is after complete extraction of the polypeptide by p97, but prior to its degradation. We can capture dislocation intermediates at this stage by expression of the EBV-DUB and observe increased association of TCRα with BAT3 under these conditions. In addition, BAT3 associates with the proteasome [25], opening the possibility that it shuttles dislocated proteins to the proteasome, akin to proteasomal shuttling factors such as RAD23 [28]. The second moment is prior to substrate engagement by p97. P97 can engage a protein ready for extraction through its cofactors UFD1 and NPL4, both of which recruit p97 to poly-ubiquitylated proteins [6], [7], [8]. However, the active domains of the E2 and E3 enzymes responsible for substrate ubiquitylation face the cytoplasmic side of the ER. Ubiquitin conjugation to the polypeptide chain thus requires the substrate to at least peek out of the ER prior to engagement of p97. This window is then extended, as substrates require de-ubiquitylation prior to their full extraction from the ER so they may pass through the central pore of p97 [20]. It is at this moment that chaperone activity could be required to allow a smooth exit from the ER and prevent a blockade induced by exposed hydrophobic domains.

## Materials and Methods

### Antibodies, Cell Lines, Constructs, and Reagents

Antibodies to Derlin1, Derlin2, SEL1L, p97 and TCRα have been described [14], [18], [21]. Antibodies against the hemagglutinin (HA) epitope tag were purchased from Roche (3F10), antibodies against BAT3 were obtained from Abcam (ab88292).

HEK293T and Hela cells were purchased from American Type Culture Collection. Cells were cultured in Dulbecco's modified Eagle's medium (DMEM), cells transduced with pLKO.1-based vectors were selected and maintained in 1 µg/ml puromycin (Invitrogen).

The Derlin2, Derlin2-GFP, HA-Ri332, p97, and TCRα constructs used for transfection experiments have all been described [12], [14]. Short RNA hairpins against either GFP or BAT3 in pLKO.1 vectors were obtained from Open Biosystems.

### In vitro transcription translation, Transient Transfection, and Viral Transduction

In vitro transciption translation essays were performed in Rabbit Reticulocyte Lysate obtained from Promega (TnT T7), as were canine microsomal membranes.

Hela cells were transiently transfected using Fugene (Roche), and 293T cells using Trans-IT (Takara Mirus Bio), according to the manufacturer's instructions. Virus production in 293T cells and viral transduction have been described [29].

### Immunoprecipitations, Pulse-Chase Experiments, and SDS-PAGE

Cells were lysed in NP40 lysis buffer (0.5% NP40, 10 mM Tris-HCl, 150 mM NaCl, 5 mM MgCl_2_, pH7.4) supplemented with a complete protease inhibitor cocktail (Roche) and 2.5 mM N-Ethylmaleimide. The immunoprecipiation was performed using 30 µl of immobilized rProtein A (IPA 300, Repligen) with the relevant antibodies, or with 12 µl anti-HA Affinity Matrix (3F10, Roche), for 3 h at 4°C with gentle agitation. The lysates were normalized to relative protein concentration prior to incubation with antibodies.

To achieve steady-state protein labeling, cells were incubated overnight with 500 µCi of [^35^S]methionine/cysteine (Perkin Elmer) in methionine/cysteine-free DMEM supplemented with 10% dialyzed IFS at 37°C.

Pulse-chase experiments were performed as described [30]. In short, prior to pulse labeling, the cells were starved for 45 min in methionine/cysteine-free DMEM at 37°C. Cells were then labeled for 10 min at 37°C with 250 µCi of [^35^S] methionine/cysteine. Incorporated radioactivity was quantified after lysis and TCA precipitation. Target protein was isolated through immunoprecipitation, and immune complexes were eluted by boiling in reducing sample buffer, subjected to SDS-PAGE (10%), and visualized by autoradiography. Densitometric quantification of radioactivity was performed on a PhosphorImager (Fujifilm BAS-2500) using Image Reader BAS-2500 V1.8 software (Fujifilm) and Multi Gauge V2.2 (Fujifilm) software for analysis.

### Immunoblotting

For immunoblot analysis, cell lysates were prepared by solubilizing cells in 1% SDS. Protein concentrations of the lysates were determined using the BCA assay (Pierce), and equivalent amounts of total cellular protein were used for immunoblotting.

### Confocal Microscopy

Cells were grown on coverslips, fixed in 4% paraformaldehyde, quenched with 20 mM glycine, 50 mM NH_4_Cl, and permeablized in 0.05% Saponin (Sigma) at room temperature. All succeeding steps are performed in the presence of 0.05% Saponin. Fixed and permeablized cells were blocked in 4% BSA and incubated either with anti-HA-Fluorescein (Roche), anti-BAT3, anti-Derlin2 or a combination thereof. Fluorophore-conjugated antibodies were obtained from Molecular Probes (Invitrogen). Images were acquired using a spinning disk confocal microscope, a Nikon 60× magnification, and a 1× numerical aperture oil lens.
